# “I will take PrEP because that’s what will help me not to get infected with HIV”: barriers to and facilitators of pre-exposure prophylaxis and condom use among adolescent girls and young women enrolled in a school-based HIV prevention program in South Africa

**DOI:** 10.3389/fpubh.2025.1616261

**Published:** 2025-11-20

**Authors:** Kate Bergh, Kim Jonas, Elona Toska, Fareed Abdullah, Nomtopi Blom, Catherine Mathews, Ngkatiseng Mthanti, Nevilene Slingers, Nathanael van Blydenstein, Zoe Duby

**Affiliations:** 1 Health Systems Research Unit, South African Medical Research Council, Cape Town, South Africa; 2 Department of Psychology, University of Cape Town, Cape Town, South Africa; 3 Centre for Social Science Research, University of Cape Town, Cape Town, South Africa; 4 Department of Social Policy and Intervention, University of Oxford, Oxford, United Kingdom; 5 Office of AIDS and TB Research, South African Medical Research Council, Pretoria, South Africa; 6 Division of Infectious Diseases, Department of Internal Medicine, Steve Biko Academic Hospital and University of Pretoria, Pretoria, South Africa; 7 Department of Public Health Medicine, Faculty of Health Sciences, University of Pretoria, Pretoria, South Africa; 8 School of Public Health and Family Medicine, University of Cape Town, Cape Town, South Africa; 9 Networking HIV and AIDS Community of Southern Africa, Pretoria, South Africa; 10 Division of Social and Behavioural Sciences, School of Public Health, University of Cape Town, Cape Town, South Africa

**Keywords:** pre-exposure prophylaxis, condoms, school-based interventions, HIV prevention cascades, adolescent girls and young women

## Abstract

**Introduction:**

The Imagine programme is a school-based HIV prevention program offering preexposure prophylaxis (PrEP), condoms and other social and structural interventions to adolescent girls and young women (AGYW) in South Africa. PrEP uptake and adherence together with the provision of condoms has not been extensively studied in the school context. We explored the barriers to and facilitators of PrEP and condom usage among Imagine programme beneficiaries using the HIV prevention cascade framework.

**Methods:**

Sixteen AGYW aged 16–20 years who had never taken PrEP, were on PrEP or had stopped PrEP were interviewed between November 2023 and March 2024. Interviews were audio-recorded, and transcripts were deductively coded according to the HIV prevention cascade steps: (1) Knowledge, (2) Motivation, (3) Access and (4) Effective use.

**Results:**

HIV and pregnancy risk awareness was high. For condom use, the risk of HIV transmission and pregnancy was outweighed by fear of sexual or physical violence from male partners and a desire to maintain relationships, as asking to use condoms demonstrated a lack of trust. High levels of PrEP knowledge motivated participants to use PrEP, especially if their partner refused to use condoms. Fear of side effects and daily pill taking were barriers to PrEP uptake. PrEP and condom services in school were highly acceptable, while anticipated stigma remained a challenge at the community clinic.

**Discussion:**

Barriers to condom use persist in South Africa, but positive attitudes toward PrEP described in this study suggest that opinions about PrEP are still forming and can be strongly influenced by youth-friendly HIV programming in schools.

## 1 Introduction

HIV prevalence in South Africa has decreased from 14.0% in 2017 to 12.7% in 2022 (1). This decline is due to a reduction in the number of new HIV infections, people dying of AIDS-related health challenges and more children being born HIV-negative (1). However, the impact of the HIV epidemic among young people aged 15 years and older is unequal across geographic areas and populations, disproportionately affecting adolescent girls and young women (AGYW) aged 15–24 years (1). In 2022, HIV prevalence among AGYW was approximately double that of their male counterparts, while in 2017, HIV incidence was reported as three times higher (1, 2). Combination HIV prevention programs offering biomedical, behavioral and structural interventions are needed to improve uptake of HIV prevention services among AGYW and prevent new infections (3).

In 2023/24, biomedical HIV prevention interventions available to AGYW in South Africa through the public health sector included oral pre-exposure prophylaxis (PrEP) and male and female condoms. Injectable PrEP and the Dapivirine vaginal ring were only offered through some HIV prevention programs and clinical trials. Oral PrEP is a tablet that can be taken daily or on-demand to prevent HIV only, while male and female condoms are barrier methods that can prevent HIV infection, pregnancy and other sexually transmitted infections (STIs). National guidelines recommend that AGYW use condoms concurrently with PrEP to prevent pregnancy and other STIs (4, 5).

PrEP is a relatively new HIV prevention method that became available in South African health facilities in 2016 (6). Barriers to PrEP uptake among adolescents and young people (AYP) in the sub-Saharan African context include inadequate PrEP knowledge, doubts about PrEP efficacy, disliking pills, a fear of side effects, PrEP stigma linked to PrEP’s association with antiretrovirals and assumptions of promiscuity, drug stock-outs, clinic waiting times, negative healthcare worker (HCW) attitudes, the complex timing of taking PrEP, parental influence and having no sexual partner (7, 8). A systematic review of PrEP delivery models for AGYW in sub-Saharan Africa suggested that AGYW may prefer school-based PrEP delivery (9), and one study among AYP in South Africa reported that uptake of PrEP was higher in schools compared to clinics and community-based youth zones (10). In addition, convenient access to PrEP and adolescent-friendly sexual and reproductive health (SRH) counseling have been highlighted as key facilitators of PrEP adherence in South Africa (11). Thus, there is a need to explore the barriers to and facilitators of PrEP uptake and adherence in the context of school-based interventions in South Africa.

The factors associated with condom use have been extensively studied since the 1990s. Among AYP in sub-Saharan Africa, these include knowledge of HIV status, a belief that condoms reduce sexual pleasure, a desire to demonstrate love and trust through condomless sex, stigma associated with promiscuity, accessibility, self-efficacy to use condoms, type of relationship (casual, faithful, transactional, age-disparate) and gendered power dynamics (12–17). However, some of these studies also emphasized the importance of understanding contextual and environmental factors when interpreting barriers to condom use, in consideration of the way in which AGYW are influenced by sociocultural norms (14, 16).

### 1.1 The unifying framework

**Figure 1:**
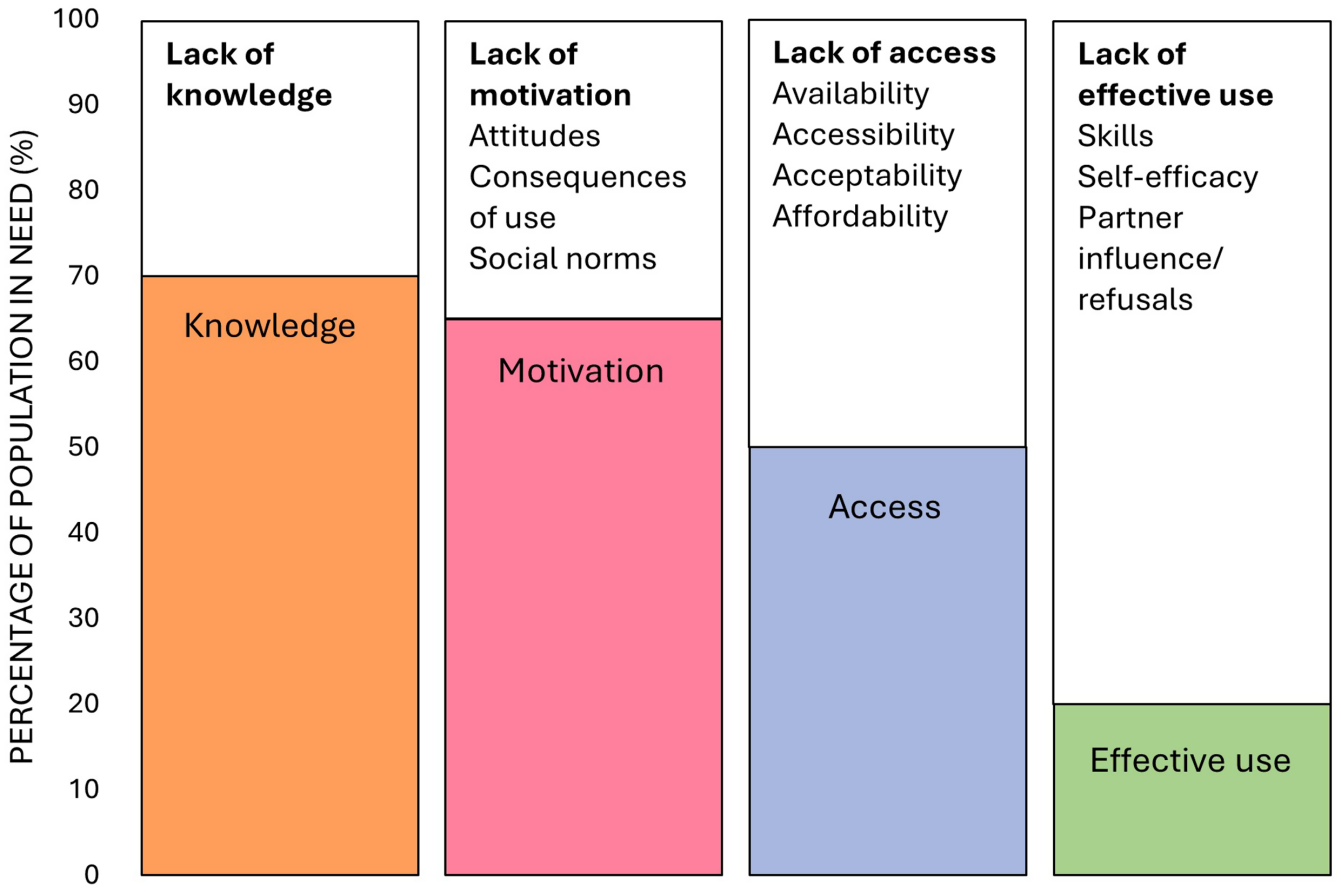
An adapted unifying framework describing knowledge, motivation, access and effective use of HIV prevention methods and the broad barriers to each step of the cascade (19), modified based on Bergh et al. findings (13). The percentages depicted in the figure are fabricated to illustrate the steps of the unifying framework.

The HIV prevention cascade is a tool used to measure the steps required by an individual in need of HIV prevention to achieve effective use of an HIV prevention method (18, 19). The unifying framework is a conceptualisation of the HIV prevention cascade which is appropriate for use in low-resourced settings with limited access to data systems (19). The cascade has three steps: motivation to use, access to and effective use of HIV prevention methods, which integrates the steps that need to be taken by both the user and provider to ensure that the prevention method is used effectively. It has also been proposed that knowledge of the intervention may be a prerequisite step that should be included in an adapted unifying framework (see Figure 1). The framework also provides an explanatory component which outlines the broad barriers to each step of the cascade (see Figure 1).

The unifying framework is grounded in behavioral and multilevel (social and structural) theories, but there is limited empirical evidence to support the theory underpinning the steps of the cascade and broad barriers to each step in low-resourced settings (13, 19–28). There is even less qualitative research that contextualizes the barriers to each step for different HIV prevention methods, population groups and geographic areas (23, 25, 27). Only one study focused on PrEP use among AGYW, but in the Zimbabwean context (23). This study qualitatively explores the perspectives and experiences of barriers and facilitators to using PrEP and condoms among AGYW enrolled in a school-based combination HIV prevention program in South Africa to contextualize the explanatory component of the adapted unifying framework. To the best of the authors’ knowledge, this is the first study to explore the barriers to and facilitators of PrEP use in the context of a school-based SRH program among AGYW in sub-Saharan Africa.

## 2 Methods

Semi-structured interviews were conducted with school-going AGYW enrolled in the Imagine programme to explore the barriers to and facilitators of, knowledge of, motivation to use, access to and effective use of PrEP and condoms in South Africa. Since female condom use in South Africa is extremely low, we did not distinguish between male and female condoms in the interviews unless specific factors associated with either method were mentioned (29). This qualitative sub-study was nested within the larger Imagine Evaluation study, a mixed-methods process and outcomes evaluation of the Imagine programme, being conducted by the South African Medical Research Council (SAMRC).

### 2.1 The Imagine programme

The Imagine programme was rolled out in March 2023 in six high schools in Moretele in the North West Province and eight high schools in Newcastle in the Kwazulu-Natal Province in South Africa. The selected areas were chosen following guidance from the National Department of Health and Department of Basic Education as they have persistent high rates of poverty and unemployment, HIV, and adolescent pregnancy, and were not already saturated with similar interventions (30, 31). The program aims to reduce HIV infection and unintended pregnancies and increase viral suppression for AGYW living with HIV. It also aims to increase early detection and linkage with antenatal care before 20 weeks of gestation for AGYW who become pregnant. The program offers clinical SRH services, psycho-social support services and social structural services, primarily through Safe Spaces located on the school premises (referred to as ‘the Imagine clinic’ by interview participants). The program was developed by the SAMRC’s Office of AIDS and TB Research, NACOSA and other stakeholders.

### 2.2 Sample and data collection

AGYW Imagine programme beneficiaries aged 16–24 years in grades 9–12 were purposively sampled from Imagine programme schools in both study areas by Imagine programme implementers. Using a script written by the SAMRC lead researcher (see Supplementary information), implementers asked beneficiaries who had never taken PrEP, were currently on PrEP or had stopped taking PrEP (based on Imagine programme records) whether they would be willing to be contacted by an SAMRC qualitative interviewer (NB or NM) and invited to participate in the study. We attempted to contact a total of 40 program beneficiaries, but some did not answer their phone after several attempts to call them, and some were unable to attend the interview during the study period as they were busy with schoolwork.

One qualitative interviewer was based in each study area for the duration of data collection. They were both female, from communities nearby to their respective study areas and fluent in the local languages (Setswana and Sesotho in Moretele and isiZulu in Newcastle). They had no relationship with the participants prior to the interviews. Both interviewers held undergraduate degrees and had prior experience conducting qualitative research in the field of SRH, positioning them to create a comfortable environment for participants to discuss sensitive topics openly.

The lead researcher (KB) created the interview guide and conducted pilot interviews, debriefing calls with the qualitative interviewers and data analysis for this study. She has a Master of Public Health and prior experience conducting research in SRH among AGYW in South Africa. However, she did not speak the local languages of the study areas, thus did not conduct all the interviews herself.

Two pilot interviews were conducted telephonically by the lead researcher, with AGYW who were comfortable conversing in English, although one of the qualitative interviewers was present in the room with the participant to assist with interpreting when necessary (November 2023). After minor modifications to the interview guide, all other interviews were conducted in-person by the two interviewers in English and/or the predominant language of the study area (January–March 2024).

The lead researcher conducted debriefing calls with the interviewers after each interview to identify key emerging findings and assess if there were any ethical issues or reportable events. Information from the debriefing calls was recorded in a report for each interview (see Supplementary information).

Interviews followed a semi-structured interview guide, based on the explanatory component of the adapted unifying framework and covering the broad barriers to knowledge, motivation, access and effective use of PrEP and condoms described in Figure 1 (19). This interview style can help participants to broach sensitive topics that they are not used to talking about (32). Vignettes were also used to broach PrEP and condom use before asking participants about their own experiences with these products and services (see Table 1) (33).

**Table 1:** Vignettes for interview guide.

Vignette section	Narrative
Introduction	*Now I would like to tell you a story about a girl called Tumi who goes to school in your community. The story is made up, but many girls in your community may have had a similar experience. I will tell you parts of the story and then ask you a few questions after each part. Would that be okay with you?*
Condom vignette	*Tumi is a 16-year-old girl at your school. She enjoys going to school and has lots of friends at school who she trusts. Tumi also has a new boyfriend, called Thabo, who is 20, and is no longer in school. You are a close friend of Tumi. One day, Tumi comes to you during break to tell you that her new boyfriend, Thabo, wants to have sex with her this weekend for the first time. Tumi really likes Thabo, but she has never had sex before and is feeling a bit nervous.*
Condom vignette continued	*Now I am going to continue with the story. Tumi has decided that she wants to get condoms to take with her to Thabo’s house on the weekend. She does not know where to get them, so she comes to you after school for more advice.*
PrEP vignette	*Now I would like to tell you a different made-up story about Sibongile, who is also a young woman at a school in your community. Is that okay with you? Sibongile has been in a relationship with an older boy at school for some time. They have sex regularly, but Sibongile is unsure whether he has sex with other women too. Sibongile tries to encourage her boyfriend, Kagiso, to use a condom when they have sex. However, Kagiso is now refusing to use a condom during sex. Sibongile does not know what to do. She has heard of a tablet/medication called PrEP which you can take to prevent getting HIV but does not know much about it. She is your friend, and she comes to you to ask for advice.*

Interviews were between 30 min and 1 hour in length. They were audio-recorded and then transcribed and translated manually by the qualitative interviewers. Participant enrolment was stopped when the lead researcher was satisfied that data saturation had been reached as the same themes were emerging among participants who had never taken PrEP or were currently on PrEP.

### 2.3 Ethical considerations

Ethics approval for this study was granted by the SAMRC’s Research and Ethics Committee (EC045-10/2020) and the Ethics Review Committee of the Faculty of Humanities at the University of Cape Town (PSY2022-040). All participants conducted an informed consent process and provided written consent before participating in an interview. For AGYW who were under 18 years, verbal consent was first obtained from a parent or legal guardian and audio recorded. All interviews were conducted in a safe and private location such as a Safe Space or classroom on the school premises or at the participant’s home, with only the participant and interviewer present. Referral and support mechanisms were in place for participants reporting abuse or mental health issues. Participants received R150 reimbursement for their time spent in the interviews. Audio recordings and transcripts of interviews were stored on a password protected computer which only the research team can access. Participants have unique identifiers to ensure confidentiality of their interview data ─ only the participant enrolment log and consent forms link the participant to their unique identifier. Unique identifiers were replaced with pseudonyms during manuscript writing to enhance readability.

### 2.4 Data analysis

The lead researcher conducted data analysis, conferring with the qualitative interviewers and co-authors regarding key findings and interpretations. Transcripts from all the interviews conducted, including those from the pilot interviews, were included in the analysis. Pilot interview transcripts were included because similar findings emerged in these interviews and only minor changes were made to the interview guide after piloting.

Data analysis was conducted following the framework method (34). Using a deductive approach, transcripts were indexed by the lead researcher according to the themes (steps of the cascade) and subthemes (barriers to each step) of the adapted unifying framework described in Figure 1, although open coding was applied to the first few transcripts to allow for other themes to emerge. Coding and indexing were conducted in NVivo version 14 (35). We used this method for data relating to PrEP and condoms separately. As data were organized into sections, overarching themes emerged for PrEP and condoms, and data were sorted accordingly in the Results section to avoid repetition.

## 3 Results

A total of 16 AGYW participated in interviews, including the two pilot interviews (see Table 2). There were an equal number of participants in each geographical study area; 11 participants were currently on PrEP, four had never taken PrEP and one had stopped taking PrEP and not restarted. Among the 11 participants who were currently on PrEP, one had stopped and restarted PrEP. Among the four participants who had never taken PrEP, one had taken the pills home but never used them.

**Table 2:** Participant characteristics.

Pseudonym	Age (years)	PrEP status
Moretele study area		
Kgotso	16	Currently on PrEP
Refilwe	17	Never taken PrEP
Kamohelo	16	Never taken PrEP
Moeletsi	17	Never taken PrEP
Dintle	16	Currently on PrEP
Teboho	18	Currently on PrEP
Amohelang	18	Never taken PrEP (took pills home but never took them)
Dikeledi	18	Stopped taking PrEP
Newcastle study area		
Aphelele	17	Currently on PrEP
Amahle	19	Currently on PrEP
Dumisani	16	Currently on PrEP
Lindile	16	Currently on PrEP
Siyabonga	20	Currently on PrEP (stopped and restarted again)
Londiwe	19	Currently on PrEP
Buhle	19	Currently on PrEP
Sanele	18	Currently on PrEP

Perspectives and opinions of participants on the barriers to and facilitators of PrEP and condom use are described below by each cascade step: knowledge of, motivation to use, access to and effective use of the HIV prevention method.

### 3.1 Knowledge

#### 3.1.1 Accuracy of information about PrEP

Almost all participants knew that PrEP is “*a pill that prevents you from getting…HIV”* (Dintle) and must be taken “*once a day”* (Teboho) at the same time every day, but some participants thought that PrEP prevents other STIs too: “*To prevent diseases before sexual intercourse you take them*” (Dikeledi). While most participants knew that PrEP has to be taken before you have unprotected sex, only some knew that PrEP has to be taken for seven days before it becomes effective. In response to one of the vignettes, most participants explained that if their hypothetical friend, Sibongile, wanted to get PrEP, she would first have to do a blood test, which some participants explained was an HIV test and others said was to make sure that “*PrEP will not give her any challenges”* (Dumisani).

If participants forgot to take their PrEP pill, some explained that they knew to take it the next day*: “I do forget but the following day I will ensure that I take my PrEP”* (Amahle), while others were not sure what they were supposed to do in that situation: “*Well about that I do not actually know how…but I have to take it there’s no …other way”* (Teboho).

Having correct and complete knowledge about PrEP motivated participants to use PrEP. Teboho described how she started using PrEP because the Imagine nurses explained “*everything about it*” to her and made her “*understand everything… related to…PrEP.”* Aphelele said that she would continue to use PrEP because she had more information about PrEP than her friends and knew that it was protecting her against HIV infection: *“because I know more about PrEP [than my friends], I will continue using it…because I’m protecting myself…I cannot listen to them [friends] because they do not know about PrEP.”*

#### 3.1.2 Access to information about PrEP

Regarding sources of information, most participants reported getting information about PrEP from the Imagine clinic but said that other sources would be the community clinic, the hospital, nurses, doctors, teachers, social workers, friends, parents and siblings. While there was importance placed on the trustworthiness of these sources of information, there was also emphasis on whether these sources had adequate knowledge about PrEP: “*She [Sibongile] can go to the community clinic or go to somebody she trusts and who is knowledgeable about PrEP”* (Londiwe). Medical practitioners were thought to be a knowledgeable source of information. Awareness raising campaigns were suggested as a way of improving knowledge about PrEP: “*We can maybe do… like at Imagine they would call us and set a tent for Imagine and only girls should come, and we also call community but only women…and explain PrEP to them, what it helps with, so that they can also not get diseases”* (Moeletsi).

#### 3.1.3 Myths and misconceptions about PrEP

Friends, family and community members were reported to not know much about PrEP unless they were friends with learners at one of the Imagine schools or also at one of the Imagine schools. Those who did have knowledge about PrEP seemed to be more accepting of it: “*Some members of my family know about PrEP so they do not [have a] problem*” (Aphelele). However, some family and community members did not believe that PrEP prevents HIV: “*I realised that this person [my mother] does not believe in this kind of thing”* (Amohelang), thought that it was an antiretroviral (ARV) for HIV treatment: “*they take it as the same as ARVs…they think when you are taking PrEP you are also affected by HIV”* (Dintle), or thought that *“PrEP is used to spread HIV”* (Buhle).

#### 3.1.4 Accuracy of information about condoms

Participants knew that condoms are used to prevent pregnancy and other diseases, often mentioning HIV and STIs. Some participants also knew that there are both male and female condoms, but most participants could not explain how condoms worked when probed further, except to say that they are not 100% effective: “*I do not know much about condoms…I just know that apparently there’s [a] female one and [a] male one, and…they are not 100% safe…they can burst during sex”* (Dikeledi).

Participants seemed to have more information about female condoms than male condoms, but did not seem to have much experience actually using female condoms: “*The male condom is the one that…the boy is supposed to put, although I have no idea how they put it… The female condom is put by the woman in her vagina, she is doing it herself before she can have sex”* (Siyabonga). Some participants were aware that the female condom was safer than the male condom because it is less likely to burst: “*The female condom is bigger so she can be protected unlike the male condom because they say male condoms burst”* (Buhle). Only Sanele expressed knowledge and understanding about how to use a condom effectively: “*the boy must put the condom and [you] will need to ensure that he has put the condom properly before having sex…[you] can see when they are done, whether it had burst or not.”*

#### 3.1.5 Access to information about condoms

When asked about sources of information about condoms, most participants suggested that you could get information about condoms from the Imagine clinic, the community clinic or a friend. Other sources of information included sisters, parents, guardians, teachers, Life Orientation classes at school, community health workers, social workers or the internet. Participants only considered talking to these sources if they trusted them as there was a fear of being judged or getting in trouble when going to the community clinic to get information and talking to your family about condoms.

### 3.2 Motivation

#### 3.2.1 HIV and pregnancy risk perception

Participants had a high-risk perception for both HIV and pregnancy, sometimes also mentioning the risk of getting STIs. When Amahle was asked why the vignette character, Tumi, would be nervous about having sex with Thabo for the first time, she replied: “*She does not know whether he has HIV or not. She is afraid that her boyfriend might infect her with the disease or get her pregnant.”* Motivation to prevent HIV infection and pregnancy also originated in having witnessed other young women who fell pregnant or acquired HIV and wanting to avoid ending up in the same situation: “*We used to see people that are sick, so I do not want to get sick…Their lives are not good at all”* (Buhle). Fear of HIV infection motivated participants to use PrEP: “*I will take it because that’s what will help me…not to get infected with HIV”* (Moeletsi), while fear of HIV infection and pregnancy motivated participants to use condoms: “*I can use the condom because I want to protect myself. I will try by all means to protect myself”* (Buhle).

Among participants who had never taken PrEP, some reported that they would only consider using PrEP if they were sexually active: “*I did not take it…Because I do not have sex”* (Refilwe), if they did not trust their partner to be faithful, or if their partner was living with HIV: “*I’ll consider using it if my partner is positive”* (Kamohelo). Among participants who were on PrEP, being sexually active was their main motivation for starting PrEP and facilitated continuation on PrEP: “*I did not trust my boyfriend…I found out last year that he has a baby that I did not know about…I felt safe because I knew that even if my boyfriend can do things behind my back, I will be safe when I am using PrEP”* (Lindile).

Amohelang, who had taken the PrEP pills home, said that she was motivated to start taking PrEP because she was in a sexual relationship, but never took the pills because her relationship ended, and she became busy with schoolwork: *“I was in a relationship…so that relationship we used to have sex, so I decided okay let me take PrEP so that I can…protect myself…Then my life started to be hard with school work so I decided to end that relationship…before I could start taking PrEP.”*

In participants’ views, having sex with an older male partner was associated with higher HIV risk because older men are more likely to have had multiple sexual partners than younger men and thus more likely to be living with HIV: “*Because he [Thabo] is older than her [Tumi]…And she does not know his past, she does not know what he was doing before her… she does not know whether [he] is sick or not”* (Refilwe).

Some participants felt that even if they were not sexually active, they were at risk of getting HIV from rape or accidents and, according to one of them, by sharing sweets or toothbrushes, thus encouraging them to take PrEP: “*PrEP is so helpful even if a person is not sexually active…For example, if you are involved in [a] car accident, you are likely to be infected because there might be blood around…We used to share sweets and if maybe the person has…bleeding gums so you are likely to be infected…If somebody decides to use my toothbrush, I will know that I will be safe”* (Siyabonga). Participants felt that AGYW like them were at high risk of being raped which could lead to HIV infection and pregnancy: “*PrEP is helpful because others are raped so the rapist might be HIV positive, so if the girl is using PrEP, she will then be protected*” (Amahle).

#### 3.2.2 Fear of side effects

Participants knew that one could experience side effects when you first started taking PrEP: “*It does have side effects. At the beginning you might feel sleepy and feel like you can vomit. There are also changes to your appetite”* (Siyabonga). The side effects that participants had heard of or experienced themselves included headaches, drowsiness, dizziness, changes in appetite, nausea, vomiting, diarrhea, fainting and kidney damage.

Fear of side effects was mentioned as a barrier to starting PrEP among participants and their friends who had never taken PrEP. Amohelang, who had even taken PrEP home with her, was too afraid to use it for fear of side effects: “*Since I collected them, I never used them because I’m scared of side effects.”* However, side effects were not reported as a barrier to continuing on PrEP among participants who were already on PrEP.

Some participants also mentioned side effects to using condoms, including that condoms were not good for men, “*causing dangers to them”* (Amahle), and could give women a “*rash”* (Refilwe).

#### 3.2.3 Disliking daily pill taking

Participants and their friends did not like the idea of taking a PrEP pill every day, especially if they were not sexually active: “*I thought it was like a burden for me to take it, like a pill every day”* (Refilwe). In addition, Moeletsi reported that she would rather use condoms because of the potential consequences of missing your PrEP pills. In contrast, participants on PrEP were motivated to take PrEP even if they disliked pill taking to prevent HIV: “*The only challenge might be to take pills every day, but it is a must that she gets PrEP so as to protect herself”* (Amahle).

#### 3.2.4 Social norms about condoms

Participants explained that friends and other young people say that sex is not enjoyable with a condom: “*Youth of nowadays we talk about issues such as condoms…they do not enjoy sex when they are wearing condoms”* (Amohelang), and that men in particular do not enjoy sex with a condom. When Dikeledi was asked how the hypothetical vignette character, Thabo, would react when asked to use a condom, she replied: *“boys they do not like condoms, they hate condoms so I do not think he will agree…because… sex is not enjoyable with [a] condom.”* Some participants even expressed a fear that men could remove the condom during sex because sex is not enjoyable with a condom: “*They say boys took out the condom intentionally during sex”* (Siyabonga).

### 3.3 Access

#### 3.3.1 Availability and accessibility of PrEP services

Most participants reported that PrEP was available at the Imagine clinic and/or the community clinic. Some participants also mentioned that one could get PrEP from a hospital or a doctor. However, in Moretele, stock-outs at the community clinic were raised as a concern, but not at the Imagine clinic: “*sometimes at the [community] clinic they run out of medicines…because at the Imagine clinic when they do not have medicine…someone will bring the medicine the very same day”* (Dintle).

Siyabonga explained how she stopped taking PrEP when she moved to Newcastle because she did not think they had PrEP in Newcastle, but then she started taking it again when the Imagine clinic came to her school: “*I stopped taking it because I thought PrEP was not available in Newcastle…I always have thoughts of going to the clinic to ask about PrEP…The safe space arrived then I continued to take PrEP.”* She also expressed uncertainty about the availability of PrEP in other parts of South Africa: “*This is my last year here at school and I am planning on going to Zululand so I wish I can get the place where I can access it so that I can continue using it.”*

PrEP was more easily accessible from the Imagine clinic compared to the community clinic because the Imagine clinic is at the school and you could go there during your break time while the community clinic is often busy: “*She [Tumi] can get it here at school because we have a clinic. She can come during break time and talk to the nurses…It will be easy at the school clinic…compared to the community clinic”* (Dumisani). However, Dikeledi said that she stopped taking PrEP because she was so busy with schoolwork that she did not even have time to go to the clinic at her school.

Some participants mentioned that it would be challenging for non-Imagine school learners to access PrEP as “*They cannot miss school just because they wanted to go to the [community] clinic”* (Amahle). Amahle, who had recently completed high school, was unsure how she could get her PrEP pills now that she was no longer at the Imagine school: “*I have not figured…out …where I will access PrEP since I am not going to school…There is no other place that I know that is providing PrEP other than…Imagine…I do not know what to do, because I do not have a number for the nurse…I will need to go to school to ask for PrEP.”*

#### 3.3.2 Availability and accessibility of condoms

All participants knew one or more places where condoms were available. Most participants said that condoms were available from the Imagine clinic or the community clinic, but some participants also mentioned shops, hospitals and libraries.

Participants described how there were always condoms at the Imagine and community clinic, but that there was not always availability of condoms in shops: “They *make sure that people who come to the [community] clinic if they want them they can take them [condoms]… sometimes they are not there [at the shops] unless maybe somewhere else but around here at our place they normally do not have condoms”* (Moeletsi).

The different locations where condoms were available seemed to provide different condom options. Moeletsi mentioned how shops only sold “*Trust”* condoms and not “*Max”* which appeared to be her preferred option. Siyabonga mentioned that both male and female condoms were available at the Imagine clinic, hospitals and in “*town*,” but that the community clinic only stocked male condoms because, according to a nurse that she spoke to: *“women do not come to take female condoms and they stay there for a long time.”*

While most participants described how condoms were easily accessible at the community clinic as one could just take them from the reception without asking, Dintle expressed concern that “*Sometimes you might not find them and…you will have to ask for a nurse to come and give you.”* At the shops, age was a concern for Lindile who explained that “*It is difficult to access them [condoms] at the shops because you will need to be 18 years and older to be able to get them.”*

#### 3.3.3 Affordability of condoms

Participants noted that condoms were free at the Imagine and community clinics. Some participants said that condoms were also free in shops, but most said that you had to buy condoms at the shops: “*At the shops also they do not ask you any questions… It will be easy because they will just sell them to her.”*

#### 3.3.4 Acceptability of Imagine clinic services

Services at the Imagine clinic were highly acceptable to participants. At the Imagine clinic, it was easy for AGYW to get PrEP and condoms because the staff were *“friendly”* (Dikeledi), *“open and free”* (Siyabonga), and “*do not judge”* (Dikeledi). They talk to the learners, provide instructions and counseling on how to use PrEP and condoms, and encourage them “*to take condoms, and other preventions”* (Dikeledi). Other learners also take PrEP and condoms from the Imagine clinic, so it has become socially acceptable, and no one asks you any questions. Amohelang explained that “*You just arrive and say you are asking for condoms. It is something that is normal…Many people do it here, so you will not actually be embarrassed, because each and every one does that”* and “*In our school it’s a normal thing [taking PrEP]…You can say it happens monthly…people especially when they leave they do not hide them [PrEP pills], a person would be holding them, so we are used to it…that people take PrEP from here.”*

#### 3.3.5 Acceptability of community clinic services

Participants felt nervous and scared when going to get condoms from the community clinic. They said that nurses would shout at them and “*be judgmental…they will ask her questions, ‘Why, you are still young?’ and all that”* (Kamohelo). Even the security guards at the community clinic were judgemental and would sometimes not let AGYW enter. Some participants said that they left the community clinic because they were treated badly: “*she [the nurse] was so harsh with me to a point where I ran away from the clinic and went home”* (Teboho) or were refused condoms: *“They might not give them to you or maybe they tell you that there’s a shortage of them, or they do not have them”* (Dikeledi). Others said that AGYW could still get what they wanted from the clinic even if they were treated badly. Participants also resorted to lying to the nurses by saying that they were getting condoms for a friend, they were “*working on a project”* (Sanele), or they needed condoms to *“tie up their socks”* (Amahle). Participants did not have experience accessing PrEP at the community clinic.

Privacy at the community clinic was an issue and participants worried that they would see people they know, and that these people would find out that they are sexually active and tell their’ families: “*From the [community] clinic…you are assisted by this one, then they refer you to another one, so everyone knows why you are there at the clinic”* (Refilwe). Some participants complained that the clinic was too busy, also increasing the chances of seeing someone that you knew: “*It can be a bit hectic, there are so many people in the [community] clinic. There might also be people from [your] neighbourhood”* (Siyabonga).

Some participants mentioned that some of the nurses at the community clinic were nice and acknowledged that everyone is different: “*It’s depending on a nurse you find…other nurse will just shout at you and other one will give you [condoms] secretly…They are different”* (Dintle). However, Siyabonga highlighted that knowledge about PrEP may be lacking among nurses at the community clinic: “*it seemed like the nurses themselves did not have enough or clear information about PrEP.”*

#### 3.3.6 Acceptability of buying condoms at the shops

Participants were also scared to buy condoms at the shops, but more for fear that other people from their community would see them and not because the shop vendors cared if they asked for condoms. Participants seemed to be less concerned about what the vendors thought because they are often not South African and just wanted the money: “*She [Tumi] can…buy the condom at the shop because the shop owners are not South African citizens. The challenge will be if there will be other people at the shops”* (Sanele).

### 3.4 Effective use

#### 3.4.1 Skills and self-efficacy to use PrEP

Participants had different strategies to help them remember to take their PrEP pills. These included making PrEP part of their routine: *“I put my PrEP next to my cosmetics, so after taking [a] bath, I will definitely be reminded of using PrEP”* (Sanele), setting an alarm on their phone, or having a family member remind them: “*My mom knows that I am taking PrEP. She even reminds me of taking PrEP”* (Buhle).

While most participants described how these reminders helped them to take PrEP once a day, Sanele reported taking her PrEP *“two times a day.”* Only Dikeledi, the participant who had stopped taking PrEP, said that taking PrEP at the same time every day was a challenge when she first started taking PrEP: “*sometimes I would forget that I was supposed to take them at a certain time.”*

Most participants were confident that they could take PrEP the way they are supposed to because it is protecting them against HIV: “*Yes, I will be able, because it is protecting my life”* (Lindile). Some participants were confident that they could continue using PrEP: “*Yes, I can continue with PrEP”* (Londiwe).

#### 3.4.2 Partner influence on PrEP

All participants had a positive attitude toward PrEP: “*I think that PrEP is a good medicine, a good treatment”* (Kgotso), because it prevents HIV even if their boyfriend is having sex with other women and does not want to use condoms with them. When Aphelele was asked if she thought PrEP was a good method for the vignette character, Sibongile, to use to prevent HIV acquisition, she said: *“Yes, because she does not know if her boyfriend is sleeping with another woman.”* The fact that one does not have to tell their partner that they are using PrEP was another positive attribute.

Most participants said that they would not tell their male partner if they were taking PrEP because using PrEP demonstrates a lack of trust in one’s partner: “*if I…just tell my boyfriend…that I take PrEP, because he does not have AIDS, he would end it and like I would feel somehow that why did I tell him…so I will not tell anyone”* (Moeletsi). Some participants said that if one’s male partner did not want to use condoms, she should not tell him that she is using PrEP because he would not approve of PrEP either. Some participants suggested that one should tell their male partner that they are using PrEP and that their partner should also take PrEP “*so that they can be both protected”* (Buhle).

#### 3.4.3 Skills and self-efficacy to use condoms

Some participants demonstrated condom negotiation skills, and described different strategies on how they would get their male partner to use a condom with them, including explaining that it was their first time having sex, that they were *“young”* (Amahle) or that their partner has more *“experience”* (Refilwe) than them.

Some participants were confident that they could negotiate condom use with their male partner: “*I would ask him before the weekend that we should use condoms because it will be my first time having sex and I want to protect myself from getting pregnant and getting diseases”* (Lindile), and use condoms during sex: “*I have to be confident and tell my boyfriend to use condoms during sex because I am still young and I do not want to get sick or get pregnant”* (Amahle). Other participants said that if their partner refused to use condoms with them, they are confident that they would not have sex with him: “*if [he] does not want to [use condoms] then I will not because this is my health, this is my body and I have every right”* (Amohelang).

However, some participants were not confident that they could negotiate condom use with their male partner: “*Eish I cannot even have that conversation [Blushing]”* (Kamohelo) and use condoms: “*I will not be confident because I do not know anything about all that. What if [he] does something during sexual intercourse…What if [he] removes that condom?”* (Teboho). Some participants explained that their confidence and ability to use condoms with their partner would depend on their partner’s age and character as well as the power dynamic in the relationship.

#### 3.4.4 Partner influence on condoms

Some participants thought that their male partner would be okay with using condoms if he was “*responsible”* and had *“the relevant information about condoms”* (Kamohelo), or if *“he is also not ready to have children*” (Lindile). However, most participants explained that if they asked their male partner to use a condom, he would think that they did not love or trust him: “*Thabo will not feel good, he will think that Tumi does not love him”* (Lindile), suspect that he is living with HIV: “*Thabo will think that Tumi is not trusting him or is suspecting him of having HIV”* (Amahle), or that they are living with HIV.

Consequences of asking one’s partner to use a condom were that it could make him angry, sometimes leading to physical or sexual violence: *“Yoo, he can get mad at Tumi, he might even want to beat her because she has mentioned these condoms…He is going to overpower her, because boys are likely to take advantage of the girls”* (Siyabonga). Alternatively, her male partner might not want to have sex with her and even leave her: “*He will tell her that when he uses a condom he will not enjoy and then there’s no need to have sex with her”* (Amohelang).

Participants explained how the idea of having sex with an older man was intimidating, affecting the power dynamic in the relationship: “*Older people might tell young ones to do things that they do not want to do”* (Sanele). There was a risk that an older male partner may coerce or force one to have sex without a condom: *“He is old…if she goes there he will force her…to sleep with him…He can even infect her with diseases”* (Moeletsi), although older male partners may also be more understanding about using condoms.

### 3.5 Dual protection

Most participants said that they would use condoms while taking PrEP to prevent getting pregnant and getting STIs. Some participants seemed to think that using PrEP concurrently with contraception other than condoms was a better idea and did not mention the risk of STIs: “*I will not use condoms because I will be using PrEP and contraceptive methods so I will be protected from getting HIV and getting pregnant”* (Amahle). Dumisani even suggested that one should use condoms, PrEP and other forms of contraception, but more for fear that one could still get pregnant while on contraception, than for fear of getting STIs: “*I can use the condom. I am taking two-month injection contraception method, but you can still get pregnant even if you are using the contraceptive method. So, I will use condoms to protect myself from getting pregnant.”*

## 4 Discussion

This study aimed to explore the barriers to, and facilitators of PrEP and condom use among AGYW enrolled in a school-based combination HIV prevention program in South Africa to contextualize the explanatory component of the unifying framework. Findings revealed high HIV and pregnancy risk perception among participants, which motivated participants to use PrEP and/or condoms. Although most participants said that they would use condoms during sex while also taking PrEP, negative attitudes toward young women’s sexuality and anticipated stigma from HCWs and older family or community members made it difficult to access condoms while unequal power dynamics in age-disparate sexual relationships and a belief that condoms reduce male sexual pleasure were barriers to condom use. Participants considered PrEP to be an appealing alternative to condoms, if their male partner was not faithful and refused to use condoms, which was more socially acceptable to friends and family members. As with condoms, accessing PrEP at the community clinic was a challenge for school-going AGYW due to anticipated stigma and a lack of time - both of which were addressed by the presence of the Imagine programme in schools, highlighting the need for youth-friendly school-based SRH services in South Africa.

### 4.1 Knowledge

Participants in this study had low knowledge about condoms and high knowledge about PrEP because they had been educated about PrEP and encouraged to use it through an HIV prevention program at their school, and the majority of the participants had taken PrEP. A narrative systematic review of adolescent condom use in Southern Africa found that SRH knowledge was low among adolescents unless they had been involved in an SRH program at their school (15). In addition, a South African survey found that the biggest barrier to willingness to take PrEP was inadequate PrEP knowledge, highlighting the importance of knowledge as a first step in PrEP use (8).

Lack of knowledge about condoms and PrEP led to several myths and misconceptions. For condoms, a common fear, and to some extent, misconception voiced by participants was that condoms are not safe because they can burst, motivating participants to use PrEP. Concerns about condom safety, breakage and ineffectiveness are common among AYP in sub-Saharan Africa (15, 36). While condoms are 98% effective at preventing HIV infection, pregnancy and other STIs if used correctly, common errors include using expired or damaged condoms, not leaving enough space at the tip of the condom, not pushing air out from the tip, and using oil-based lubricants (37, 38). The low levels of knowledge on correct condom use among participants in this study highlights the need for instruction and counseling about condom use among AGYW, which was available through the Imagine programme, but might not have been prioritized given the emphasis on PrEP.

Regarding PrEP, family members who understood what PrEP was, were accepting of PrEP, while family and community members who did not have knowledge about PrEP, did not believe that it could prevent HIV, as described by other studies in sub-Saharan Africa (7, 13). Other myths and misconceptions included a belief that PrEP was a treatment for HIV and that PrEP was used to spread HIV; both highlighted by other studies in the region (39, 40). However, these other studies mentioned additional myths and misconceptions that did not come up in this study, including that young women who use PrEP are promiscuous, PrEP causes resistance to ARVs or is a post-exposure prophylaxis, and that PrEP causes termination of pregnancy or infertility, suggesting that the Imagine programme has created awareness and understanding about PrEP in the study communities (7, 39, 40). The Program intentionally focused on teaching AGYW to be competent and confident PrEP users, framing PrEP use as a practical life skill rather than a shameful intervention for promiscuous AGYW aiming to shift norms surrounding PrEP use.

### 4.2 Motivation

Among study participants, having a high HIV risk perception and knowing that PrEP is a pill that can prevent HIV even if one has unprotected sex and does not trust one’s partner, motivated participants to use PrEP. Qualitative studies conducted among AGYW in sub-Saharan Africa have also found that distrust of one’s partner, having a partner living with HIV, high HIV risk perception and a desire to remain HIV-negative were facilitators of PrEP uptake (7, 40). Participants in this study had a high HIV risk perception, especially in the context of age-disparate sexual relationships. Other studies in Southern Africa have also described how AGYW were aware of the risk of HIV acquisition when having age-disparate and transactional sex, but that this fear was overpowered by factors such as poverty and a desire to maintain the relationship (15).

Despite the high risk of acquiring HIV that participants described even if one was not sexually active (from rape and accidents) and efforts by the Imagine programme to encourage PrEP uptake among all AGYW who are likely to become sexually active, most participants still reported that they would only use PrEP if they were sexually active and, in some cases, if their partner was living with HIV. Among participants of a qualitative study conducted in Uganda, South Africa and Zimbabwe, having no sexual partner or having one faithful partner were barriers to PrEP uptake (7). The notion that AGYW should only use PrEP if their sexual partner was living with HIV is outdated and probably based on the World Health Organization’s initial recommendation that PrEP only be used in serodiscordant relationships, which still seemed to be recommended by HCWs according to one study in Namibia (41), although this recommendation has now been updated to include anyone at substantial risk of HIV infection (42).

Participants had very positive attitudes toward PrEP as it gave them agency to protect themselves against HIV even if there were other factors, such as those mentioned above, which put them at risk of acquiring HIV that were out of their control. Other studies in sub-Saharan Africa have also reported that AGYW associate PrEP with a happy feeling of being HIV negative, inspiring agency, empowerment and self-worth (40).

Participants and their friends were accepting of PrEP as PrEP had become socially acceptable at the Imagine schools. Participants and friends who had never taken PrEP feared side effects, but those who had ever taken PrEP knew that side effects did not last and did not report them as a barrier. Disliking daily pill taking was also reported as a barrier to taking PrEP among participants and friends who had never taken PrEP, but for participants on PrEP, this was overpowered by their fear of HIV acquisition. Fear of side effects and actual side effects have been reported as a barrier to uptake of and adherence to PrEP by other studies in sub-Saharan Africa, which recommended that HCWs clearly describe potential side effects to PrEP users before they start taking the pills, reassure them that side effects usually only last for the first month of use or suggest that they adjust the time that they take PrEP (8, 39, 40, 43). Other studies have also reported how AGYW in sub-Saharan Africa found pill taking to be a burden, especially when they were healthy; that they did not like the size, smell or taste of the pills; and found it complicated to take PrEP at the same time every day (7, 39, 40). Injectable PrEP may solve the issue of disliking pills when it becomes available as these studies also found that participants had a preference for injectables linked to their familiarity with the contraceptive injection.

PrEP was more socially acceptable than condoms to friends, family and community members, which seemed to be attributed to the positive awareness raising about PrEP by the Imagine programme. Friends described how sex was not enjoyable with a condom, while AGYW who tried talking about or obtaining condoms felt judged and stigmatized by older family and community members for having sex when they are too young, as described by other studies in Southern Africa (14, 15). While the stigma associated with condoms seems to persist in South Africa, perhaps there is an opportunity to change the narrative around AGYW sexuality in the context of PrEP use.

### 4.3 Access

While participants described how condoms were widely available, most had only ever accessed PrEP from the Imagine clinic. Accessing PrEP before the Imagine programme started in the chosen Imagine schools and once participants had left school were highlighted as a challenge, emphasizing the important role that the Imagine programme has played in creating awareness about PrEP and initiating AGYWs on PrEP but also raising questions about the sustainability and long-term benefits of school-based SRH programs unless they have clear strategies for continuing beneficiaries on PrEP after school and working with existing services in the public health sector.

PrEP stock-outs at the community clinic was raised as a concern in Moretele, while having time to access PrEP from the community clinic was a challenge amidst participants’ busy school schedule in both study areas. There is limited evidence for PrEP stock-outs in South Africa in the literature, but South African participants in one study did raise concerns about potential stock-outs possibly due to their experiences of stock-outs with other medications (7). Attending school, distance and cost of travel and waiting times at health facilities have been raised as a barrier to accessing PrEP in other studies among AGYW, pregnant and postpartum women, and men in sub-Saharan Africa (9, 44), making school services appealing. Since PrEP is an HIV prevention method given to people who are usually not sick, convenient and easy access is an important component of continuation on PrEP.

Services at the Imagine clinic were far more acceptable to participants compared to the community clinic because they were faster, more convenient and private. Several studies in South Africa have highlighted AGYW’s preference for youth-friendly access to PrEP in clinics set aside from the main health facility for adult clients, such as community-based drop-in centers or access to PrEP at school (9, 40). Safe Spaces such as the Imagine clinic have been shown to be acceptable to and popular among AGYW enrolled in a community-based HIV prevention program in South Africa, who are often attracted by educational and recreational activities at the Safe Space, but who then engage in SRH services also offered there (45). Given the ambitious clinical targets of the Imagine programme, SRH knowledge creation and service uptake were incorporated into a risk assessment game that both attracted AGYW to the Safe Space and gave them access to program services.

Staff at the Imagine clinic were considered to be less judgemental and more knowledgeable about PrEP compared to staff at the community clinic. Fear of judgment and anticipated stigma when AGYW access condoms is a widespread problem in Southern Africa (15). Both findings from this study and a study in Namibia suggest that knowledge about PrEP among HCWs in the public health sector is inadequate (41). Imagine coaches and clinical staff had all completed the National Department of Health’s PrEP online learning certificate (46). Improving timely PrEP knowledge given the fast-developing evidence and knowledge base on PrEP among HCWs is critical to encouraging uptake of PrEP among AGYW from public health facilities.

### 4.4 Effective use

Participants had different strategies for negotiating condom use with their male partners including telling them that it was their first time having sex or that they were still young and wanted to avoid pregnancy. However, having the confidence to enact these strategies and actually use condoms with their male partner was mixed and most participants did not know how to use condoms correctly. In addition, self-efficacy to use condoms was also influenced by the power dynamic in the relationship, with participants reporting that it would be more difficult to negotiate condom use with an older male partner. Self-efficacy and being in an age-disparate relationship are known barriers to condom use (15).

In contrast, participants knew how to use PrEP correctly and were very confident that they could use it in the way they were supposed to. Self-efficacy has been highlighted as an important facilitator of motivation to use PrEP among AGYW in one South African survey (13). Despite studies which reported that taking PrEP at the same time every day was a challenge (7, 39, 40), this study found that participants had several strategies to remembering to take their pills including setting an alarm reminder, making PrEP part of their daily routine and having a family member remind them to take PrEP – many of which were part of the Imagine programme content.

Participants explained that there were several risks to asking their male partner to use condoms with them including that their partner may think that they did not love or trust them, may leave them or may become physically or sexually violent. Other South African studies among AGYW have also explained how a desire to demonstrate love and commitment and maintain a relationship as well as a fear of violence were obstacles to condom use (14, 47). Additionally, participants explained that AYP disliked condoms because of potential side effects and a belief that it reduces male sexual pleasure, also highlighted as barriers in the same two studies. Taken together, these findings demonstrate two key barriers to condom use which seem to persist in South African society: unequal power dynamics and the prioritization of sexual pleasure over sexual health.

With these major barriers to condom use in mind, PrEP was a very appealing alternative to participants, as it does not require partner buy-in. Most participants said that they would not tell their male partner that they were using PrEP because it demonstrated the same lack of trust described in the context of condoms, which could negatively impact their relationship. Disclosure to a family member such as a mother was more common. A study on PrEP disclosure among AGYW in South Africa found that younger AGYW were less likely to disclose PrEP use to a partner, compared to older AGYW who lived with their partner, but that those who did, often had a positive reaction and received support from their partner (48). However, PrEP disclosure also altered perceptions of trust in their relationship, but only in some cases, raising the question of whether there is an opportunity to dissociate PrEP use with the connotations of condom use in sexual relationships. The reaction of household members to PrEP disclosure was the most important disclosure in determining PrEP adherence, as a positive response could support PrEP adherence and assist with daily pill reminders while a negative response may lead to PrEP discontinuation.

### 4.5 Dual protection

Although most participants reported that they would use condoms while on PrEP, it was clear from their lack of knowledge about condoms that this was not actually being put into practice. This could be because most participants on PrEP were also using contraception other than condoms and either did not know that PrEP does not prevent STIs other than HIV or felt that contracting STIs was a secondary concern compared to HIV infection and pregnancy. However, it could also be that the whole appeal of PrEP is that you do not have to use condoms to protect yourself against HIV. This is supported by our findings as well as a qualitative study of a PrEP service in Kenya that reported that male and female PrEP users regarded PrEP as a more feasible alternative to condoms because it did not affect sexual pleasure and conception, and would not lead to the same conflict and stigma as condoms (49). In the context of a program that provided PrEP and risk-reduction counseling to AGYW, condom use was increased among PrEP users (50), highlighting the important role that program implementers can play in encouraging dual use of PrEP and condoms.

### 4.6 Limitations

Limitations to this study included that only a small number of participants who had never taken PrEP or discontinued PrEP were recruited but the same key barriers to PrEP uptake were mentioned by most participants who had never taken PrEP and both participants who had never or ever taken PrEP provided valuable insights into potential reasons for stopping PrEP. Although not generalisable to all of South Africa and sub-Saharan Africa, these data from communities across two hard-to-reach and vulnerable study areas are valuable. Findings may be subject to social desirability bias, but this should be limited by the thorough informed consent process which explained to participants that there were no immediate benefits to participating in the study, except for a R150 reimbursement for their time. While participants may have also had difficulties expressing their opinions in English and been more likely to withhold certain confidential information during the two telephonic pilot interviews, a qualitative interviewer fluent in the predominant languages of the study areas was present to assist with interpreting during pilot interviews and all other interviews were conducted in person.

## 5 Implications and conclusions

The findings suggest that while several barriers to condom use are widespread across sub-Saharan Africa and persist in South Africa for this well-established HIV prevention method; the barriers to and facilitators of PrEP use, a relatively new HIV prevention method, may be context-specific and malleable as they were strongly influenced by the Imagine programme. South African HIV prevention programs need to act now, creating awareness and providing information about PrEP to vulnerable communities while social norms around PrEP are still forming. Access to SRH services in schools was highly acceptable to participants and we encourage further implementation of school-based programs, however, access in the public health sector was not acceptable to participants, highlighting the need to both improve these services for AGYW not benefiting from school-based programs or who have left school and offer youth-friendly services in convenient spaces outside of community clinics. PrEP delivery services need to emphasize the importance of concurrent PrEP and condom use, also providing information on how to use condoms correctly. Mothers and other household members should be included in PrEP programming so that they can support the AGYW PrEP user.

**Figure 2:**
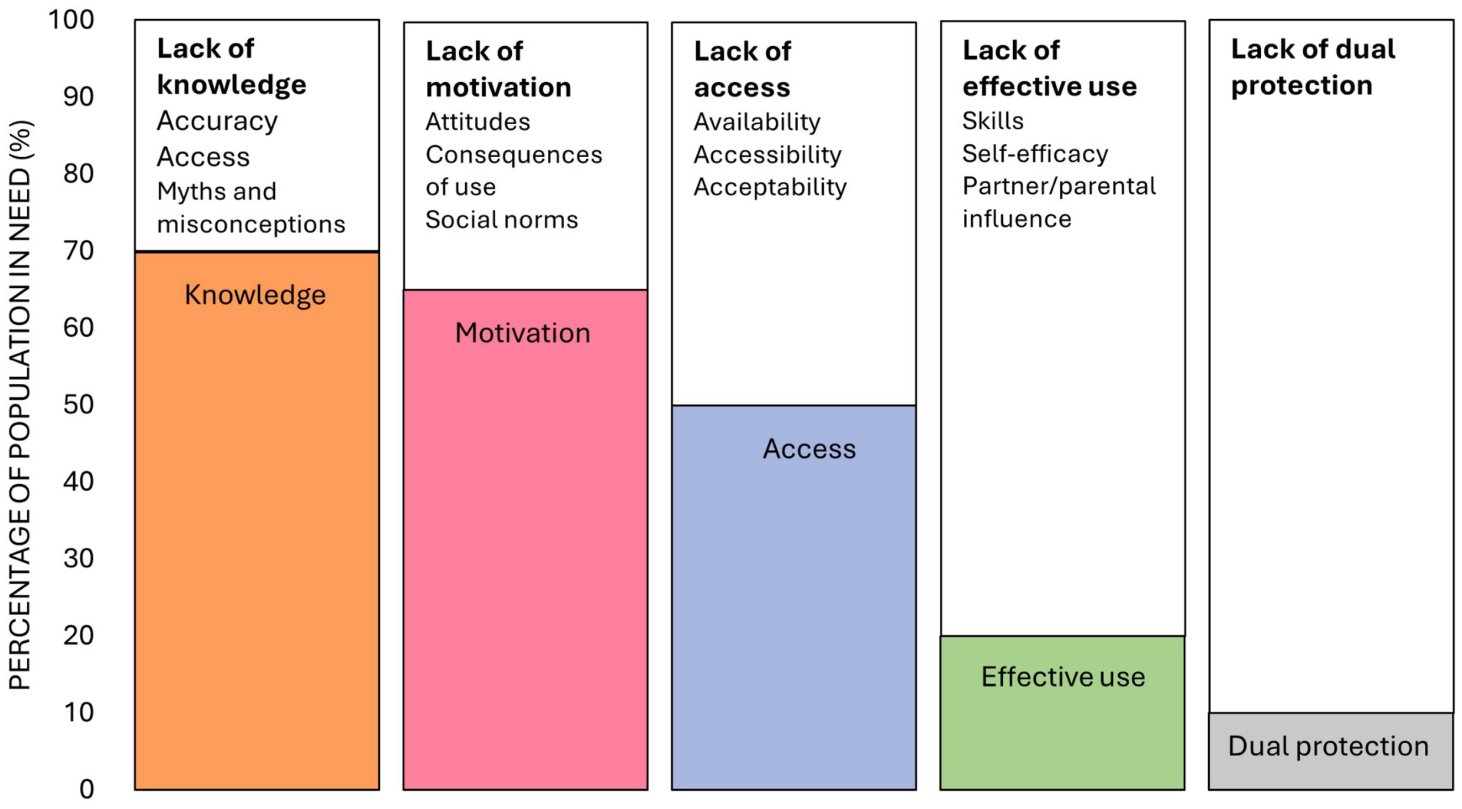
A revised unifying framework describing knowledge, motivation, access and effective use of HIV prevention methods and the broad barriers to each step of the cascade (13, 19), modified based on findings from this study. The percentages depicted in the figure are fabricated to illustrate the steps of the revised unifying framework.

In terms of the unifying framework, these findings support adaptations proposed by Bergh et al. to include knowledge as the first step of the cascade and attitudes as a broad barrier to motivation (13). We also propose additional adaptations to consider based on these findings. Firstly, we propose three broad barriers to knowledge including accuracy of information, access to accurate information sources and myths and misconceptions. Secondly, affordability may not be a relevant barrier in the context of programs or public health systems where HIV prevention methods are offered for free. Thirdly, effective use of PrEP may be more influenced by parents than sexual and romantic partners as AGYW are more likely to be living with their family than their partners. Finally, an additional cascade step may be required when researching more than one prevention method. This step should measure dual use of the HIV prevention methods and the potential barriers to using both methods. These barriers may be complex and require qualitative research to explore. These adaptations are depicted in the revised unifying framework described in Figure 2.

## Data Availability

To protect the privacy and confidentiality of the adolescent girls and young women who participated in this study, the full datasets are not publicly available, in accordance with the approved protocol. However, anonymised subsets of the data can be made available upon reasonable request. Requests should be directed to the SAMRC’s institutional ethics committee at Adri.Labuschagne@mrc.ac.za.
