# Supplementary material for: “I will take PrEP because that’s what will help me not to get infected with HIV”: barriers to and facilitators of pre-exposure prophylaxis and condom use among adolescent girls and young women enrolled in a school-based HIV prevention program in South Africa
Source: Front Public Health. 2025 Nov 20;13:1616261. doi: 10.3389/fpubh.2025.1616261 (PMC12683664; doi:10.3389/fpubh.2025.1616261)
Supplement: Supplementary file 1 [file Data_Sheet_1.PDF]

Supplementary information

S1. Script for interview referrals from the Imagine Programme

The South African Medical Research Council is conducting interviews with young women like yourself about their health and the services that you may need or have already received through the Imagine Programme. Based on your health assessment, you may be eligible for an interview. Would you like to hear about the study so that you can have an opportunity to participate?

(If yes) The study is called the Imagine Evaluation. You may have already heard about it. The Imagine Evaluation is assessing the Imagine Programme to make sure that the programme provides all the services that young women like you may need. During the interview, you may be asked about your life, your family, your school, your relationships, your health, your sexual behaviour and the services that you need or have already received. The interviews will be conducted in a safe, private and confidential space with a research team member. The interview will be recorded but your name will not be saved with the recording. Only the Imagine Evaluation research team will have access to the interview recording. You will receive a R150 ABSA CashSend reimbursement for your time.

If you would like an opportunity to participate in an interview, you can give me your number. I will share this number with the Imagine Evaluation team who will TRY TO contact you to invite you to participate in an interview. Would you be willing to be contacted by the Imagine Evaluation team to be invited to an interview? THE IMAGINE EVALUATION TEAM WILL NOT BE ABLE TO INTERVIEW ALL WILLING PARTICIPANTS AS THEY CAN ONLY INTERVIEW A LIMMITED NUMBER OF PARTICIPANTS.

Note. The text in CAPS was added midway through data collection as it became apparent that not all potential participants referred to the study from the programme would be able to be enrolled into the study and interviewed. However, the interviews tried to interview everyone who they invited to the study.

S2. Post-interview debriefing call questions for interviewers

1. How was the interview?
2. What was the participant’s behaviour and mood?
3. Where there any adverse events?
4. How was the location of the interview?
5. What were the main barriers to male condom use?
6. Had the participant ever taken PrEP?
7. What does the participant like about PrEP?

8. What were the main barriers to PrEP use?
